# Simultaneous purification of nitrile hydratase and amidase of *Alcaligenes* sp. MTCC 10674

**DOI:** 10.1007/s13205-013-0163-z

**Published:** 2013-08-27

**Authors:** S. K. Bhatia, P. K. Mehta, R. K. Bhatia, T. C. Bhalla

**Affiliations:** Department of Biotechnology, Himachal Pradesh University, Summer Hill, Shimla, 171005 India

**Keywords:** *Alcaligenes* sp. MTCC 10674, Bienzymatic, Nitrile hydratase, Amidase

## Abstract

*Alcaligenes* sp. MTCC 10674 has a bienzymatic system for the hydrolysis of nitriles. The nitrile hydratase and amidase have been purified simultaneously to homogeneity using a combination of (NH)_4_SO_4_ precipitation, ion exchange chromatography and gel permeation chromatography. Nitrile hydratase and amidase have molecular weight of 47 and 114 kDa, respectively and exist as heterodimer. Optimum temperatures for maximum activity of nitrile hydratase and amidase were 15 °C (2.4 U/mg protein) and 45 °C (2.3 U/mg protein), respectively. Nitrile hydratase showed maximum 7.8 U/mg protein at 50 mM acrylonitrile and amidase has 9.2 U/mg protein at 25 mM propionamide. Nitrile hydratase has *V*_max_ 10 μmol/min/mg and *K*_m_ 40 mM, while amidase has *V*_max_ 12.5 μmol/min/mg and *K*_m_ 45.5 mM, respectively. Heavy metal ions Hg^2+^, Ag^+^, Pb^2+^ and Cu^2+^ were strong inhibitors of nitrile hydratase and amidase activity.

## Introduction

Nitrilase (EC 3.5.5.1), nitrile hydratase (EC 4.2.1.84) and amidase (EC 3.5.1.4) constitute an important class of nitrilase superfamily (Branner 2002). Nitrilase hydrolyzes the nitriles to acids and ammonia in one-step reaction (Sorokin et al. 2007). However, nitrile hydratase first hydrates the nitrile to corresponding amide which is subsequently hydrolyzed by amidases into organic acid and ammonia (Bhalla and Kumar 2005; Bhatia et al. 2013). There is considerable industrial interest in the enzymatic conversion of nitriles because of the increasing demand for conducting such conversions under mild conditions that are often compatible with the sensitive structures of many industrially important compounds. This environmental friendly bioconversion allows clean and mild synthesis with high selectivity and yield. Several bioprocesses have already been reported for the conversion of nitriles and amide compounds into their corresponding acids (Raj et al. 2006). Number of amides has been synthesized using nitrile hydratase, e.g., nicotinamide and butyramide (Raj et al. 2006; Prasad et al. 2007). Amidases are used as catalyst in effluent treatment and their acyltransferase activity is harnessed for the synthesis of pharmaceutically important compounds such as acetohydroxamic acids and benzohydroxamic acid (Prasad et al. 2007; Sharma et al. 2012; Bhatia et al. 2012). Despite immense potential of these hydrolyzing enzymes, these are not used for the commercial production of acids because of non availability of desired enzymes vis-à-vis their cost, selectivity and stability. *Alcaligenes* sp. MTCC 10674 has a nitrile hydratase and amidase bienzymatic system for the hydrolysis of nitriles. Nitrile hydratase and amidase system was used for the production of α-hydroxyisobutyric acid from α-hydroxyisobutyronitrile (Bhatia et al. 2013), which finds its use in the synthesis of polymethyl methacrylate and acrylic glass (Singh et al. 2006) and acyltransferase activity of amidase used for benzohydroxamic acid production (Bhatia et al. 2012). Whole cell has nitrile hydratase activity accompanied with amidase activity which led to carboxylic acid as side product during the conversion of nitriles into amide (Brady et al. 2004), and purified enzyme can be used to overcome this. Therefore, the objective of present study was to develop simple steps for the purification of nitrile hydratase and amidase to reduce the cost of enzyme production and to characterize the nitrile hydratase and amidase using acrylonitrile and propionamide as substrate, respectively.

## Materials and methods

### Chemicals

All chemicals were of analytical grade and they were purchased from Alfa Aesar, Johnson Matthey Company and Sigma (India).

### Medium and cultural conditions

*Alcaligenes* sp. MTCC 10674 previously isolated from the soil sample of orchid garden of Kinnaur District of Himachal Pradesh (India), was cultured in minimal salt media (MSM) having pH 7.0 and containing g/L, Na_2_HPO_4_·12H_2_O 2.5 g, K_2_HPO_4_ 2.0 g, MgSO_4_·7H_2_O 1.0 g, FeSO_4_·7H_2_O 0.1 g, CaCl_2_·2H_2_O 0.6 g, peptone 5.0 g at temperature 25 °C for 24 h. MSM was supplemented with 40 mM isobutyronitrile after 6 h of growth as nitrogen source.

### Enzyme assay of nitrile hydratase and amidase

#### Nitrile hydratase assay

The assay mixture contained 0.125 M NaH_2_PO_4_/Na_2_HPO_4_ buffer (pH 8.0), 50 mM acrylonitrile and purified nitrile hydratase of *Alcaligenes* sp. MTCC 10674 at 15 °C for 20 min. Acrylamide production was measured spectrophotometrically at 230 nm. One unit of nitrile hydratase activity was defined as amount of enzyme that hydrates the acrylonitrile to release 1 μmol of acrylamide per minute under assay condition.

#### Amidase assay

Assay of purified amidase was carried out in 0.075 M NaH_2_PO_4_/Na_2_HPO_4_ buffer (8.0), 50 mM amide and purified amidase of *Alcaligenes* sp. MTCC 10674 at 45 °C for 20 min. Ammonia assay was performed for the amidase activity. One unit of amidase activity was defined as the amount of enzyme that hydrolyzes the propionamide to release 1 μmol of ammonia per minute under assay condition. Protein estimation was done according to Bradford method (Bradford 1976).

### Purification of nitrile hydratase and amidase

#### Preparation of cell free extract

*Alcaligenes* sp. MTCC 10674 cells were cultured in 1 L minimal salt medium at 25 °C. After 24 h of incubation, cells were harvested from the culture broth by centrifugation at 10,000 *g* and washed twice with 0.1 M K_2_HPO_4_/KH_2_PO_4_ (pH 7.0) and suspended in the same buffer. Bacterial culture of *Alcaligenes* sp. MTCC 10674 (15 mg/ml) was disrupted using BeadBeater™. The resultant suspension was centrifuged at 10,000 g at 4 °C for 20 min to remove cell debris. The supernatant fluid was designated as the cell free extract (cell lysate) and stored at 4 °C.

#### Ammonium sulfate fractionation

The cell free extract was subjected to ammonium sulfate saturation (20–60 %) and the precipitates collected after centrifugation at 15,000 g (25 min at 4 °C) were suspended and dialyzed against the same buffer. The ammonium sulfate fractionate (ASF) having nitrile hydratase and amidase activity was further used for purification.

#### Ion exchange chromatography

The ASF having nitrile hydratase and amidase activity were subjected to DEAE-ion exchange chromatography. After loading the ASF onto the column, it was washed with potassium phosphate buffer pH 7.0 (0.05 M), until there was no further elution of protein. The column was subsequently eluted with a linear gradient of NaCl (from 0 to 0.5 M) in the same buffer.

#### Gel permeation chromatography

The protein fraction of ion exchange chromatography having amidase activity were pooled together and applied to gel permeation chromatography column. The gel permeation chromatography was performed using column (2.6 × 60 cm) packed with Sephacryl S-100 high resolution (GE Healthcare) matrix. The gel permeation column was pre-equilibrated with buffer and it was eluted with potassium phosphate buffer pH 7.0 (0.05 M) at a flow rate of 1.0 ml/min. The molecular weights of purified nitrile hydratase/amidase of *Alcaligenes* sp. MTCC 10674 were determined by SDS/Native-PAGE (Laemmli 1970).

### Characterization of purified nitrile hydratase/amidase

#### Buffer pH

The activities of purified nitrile hydrolyzing enzymes of *Alcaligenes* sp. MTCC 10674 were assayed in different buffers, e.g., citrate buffer (4.0–6.0), sodium phosphate buffer (6.0–8.0), potassium phosphate buffer (6.0–8.0), borate buffer (7.0–9.0) and carbonate buffer (9.0–10.0). The 1.0 ml reaction mixture contained 0.1 M of specified buffer, and different substrates, acrylonitrile for nitrile hydratase and propionamide for amidase. Reaction was carried out at 30 °C for 1 h.

#### Buffer molarity and temperature

The activity of purified nitrile hydrolyzing enzyme was estimated in K_2_HPO_4_/KH_2_PO_4_. The effect of buffer molarity on the activities of the enzymes was studied by varying the buffer concentration from 0.025 to 0.125 M in the reaction. The temperature optimum was determined by varying reaction temperature from 25 to 55 °C.

#### Incubation time and stability

Incubation time for optimum activity of nitrile hydrolyzing enzyme was studied by varying the incubation time of reaction from 10 to 90 min. Thermal stability of the purified enzymes (nitrile hydratase/amidase) was investigated at 25 to 55 °C.

#### Effect of metals ion and other chemicals

The nitrile hydratase and amidase activity was assayed in the presence of various metal ions and chemicals (CaCl_2_, CdCl_2_, CsCl_2_, CoCl_2_, CuSO_4_, FeSO_4_, HgCl_2_, MgSO_4_, MnCl_2_, ZnSO_4_, KCl, NaCl, EDTA, DTT and urea) with a final concentration of 1 mM.

## Results and discussion

### Purification

**Fig. 1:**
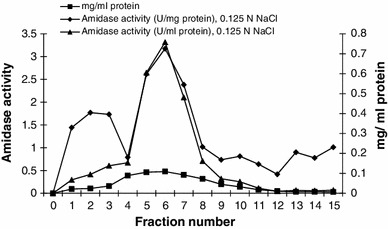
Elution profile of protein and amidase activity with 0.125 N NaCl during DEAE-Sepharose chromatography

**Fig. 2:**
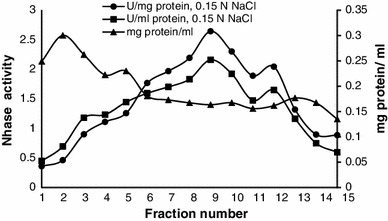
Protein elution profile and Nhase activity profile in ion exchange chromatography with 0.15 N NaCl elution

**Fig. 3:**
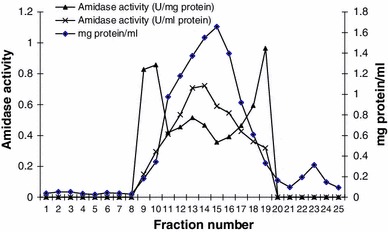
Elution profile of protein and amidase activity during gel permeation chromatography

**Fig. 4:**
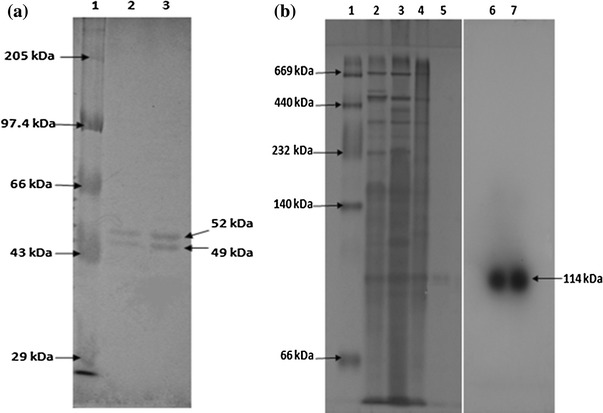
**a** SDS-PAGE of purified fractions of amidase, lane (1) marker (2) and (3) purified fraction. **b** Native-PAGE analysis of purified amidase fraction, lane (1) Marker (2) crude (3) precipitate (4) pooled fraction of ion exchange chromatography (5) purified fraction of gel permeation (6) and (7) zymogram of purified fraction 6

**Fig. 5:**
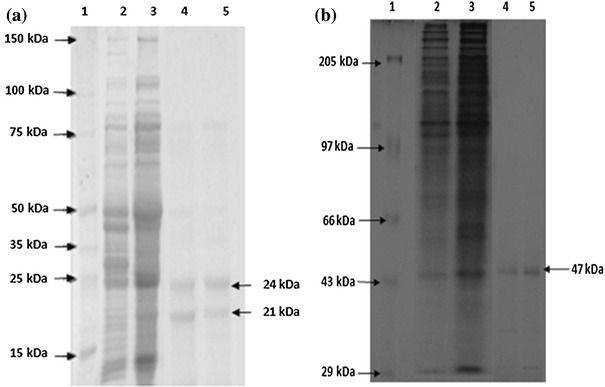
SDS-PAGE of nitrile hydratase, **a** lane (1) marker (2) crude (3) precipitate (4) fraction number 14, 15. **b** Native-PAGE analysis of purified nitrile hydratase (1) marker (2) crude (3) precipitate (4) purified fraction 14 and 15

Nitrile hydratase and amidase of *Alcaligenes* sp. MTCC 10674 were purified using different chromatography techniques. Acrylonitrile was used as substrate for nitrile hydratase characterization and propionamide was used as substrate for characterization of amidase. DEAE Sepharose ion exchange chromatography was performed and amidase got eluted with 0.125 M NaCl in fraction number 4–9 (Fig. 1) and further increase in NaCl concentration up to 0.15 M resulted in nitrile hydratase elution (Fig. 2). Fraction number 6, 7, 8, 9, 10 and 11 of 0.15 M NaCl elution contained purified nitrile hydratase and 6.3-fold purification was achieved in a single step (Table 1). Nitrile hydratase has been already purified from various organisms *Corynebacterium pseudodipthereticu* and *Rhodococcus rhodochrous* PA-34 up to 8.8 and 52-fold, respectively (Li et al. 1992; Prasad et al. 2009). Fraction 4, 5, 6, 7 and 8 obtained with 0.125 M NaCl showed amidase activity, were pooled together and applied in gel permeation column for further purification. Purified amidase was obtained in fraction number 14, 15 and 16 (Fig. 3), and 9.2-fold purification was achieved (Table 2), previously amidase has been purified from *Pseudonocordia thermophila* and *Delftia tsuruhatensis* CCTCCM 205114 up to 48 and 105-fold, respectively (Egorova et al. 2004; Wang et al. 2011). The purified amidase consists of two subunits of 52 and 49 kDa (Fig. 4a), while in native PAGE a single band of 114 kDa was observed (Fig. 4b). Nitrile hydratase also exists as heterodimer of 24 and 21 kDa (Fig. 5a), and in native PAGE a single band of 47 kDa was observed (Fig. 5b). Purified amidase exists as dimer (52 and 49 kDa) and has two subunits α and β as the amidase of *Pseudonocardia thermophila* (2*52 kDa), *Brevibacterium* sp. R312 (2*54.7 kDa) (Egorova et al. 2004; Baek et al. 2003). Nitrile hydratase was found as heterodimer of 24 and 21 kDa, as already reported in *Rhodococcus* sp. AJ270 (α-22.9, β-23.4) and *Rhodococcus equi* TG328-2 (α-23, β-24) (Song et al. 2007; Rzeznicka et al. 2010).

**Table 1:** Purification summary of nitrile hydratase of *Alcaligenes* sp. MTCC 10674

Purification step	Volume (ml)	Protein (mg/ml)	Total protein (mg)	Total activity (U)	Specific activity (U/mg)	% Yield	Fold purification
Homogenate	40	3.3	132	47.2	0.35	100	1
20–60 %	12	6	72	35.0	0.48	74	1.4
DEAE-Sepharose	8	0.163	1.304	2.904	2.22	6	6.3

**Table 2:** Purification summary of amidase of *Alcaligenes* sp. MTCC 10674

Purification step	Volume	Protein (mg/ml)	Total protein (mg)	Total activity (U)	Specific activity (U/mg protein)	% Yield	Fold purification
Homogenate	40	3.3	132	18	0.14	100	1.0
20–60 %	12	6	72	12	0.16	66	1.1
DEAE-Sepharose	10	1.4	14	5.5	0.40	30	2.9
Sephacryl-100	6	0.44	2.64	3.36	1.30	19	9.2

### Buffer pH and molarity dependence of Nhase and amidase

Nhase and amidase have maximum hydrolysis activity, i.e., 1.75 U/mg protein and 1.2 U/mg protein, respectively in sodium phosphate buffer (pH 8.0). Nitrile hydratase requires 0.125 M buffer for optimum activity (0.14 U/mg protein) and maximum amidase activity was achieved in 0.075 M (1.95 U/mg protein). Both the enzymes are active at neutral pH, as already reported for the nitrile hydratase of *Corynebacterium* sp. C5 (Tani et al. 1989).

### Effect of temperature

Amidase showed maximum activity at 45 °C (2.3 U/mg protein), while nitrile hydratase at 15 °C (2.4 U/mg protein). Deviation from the optimal temperature decreased the activity of both enzymes. Amidase has optimum activity at 45 °C same to the amidases of *Pseudonocardiathermophila*, *Bravibacillus* sp. R312, and *Sulfolobussolfataricus,* while hydratases require low temperature for its optimum activity as already reported in *Brevibacterium* sp. R312, *Rhodococcus* sp. AJ270 and *Rhodococcusequi* TG328-2 (Egorova et al. 2004; Baek et al. 2003; Song et al. 2007; Rzeznicka et al. 2010; Kotlova et al. 1999; d’Abusco et al. 2001).

### *K*_m_ and *V*_max_ value

Amidase has *V*_max_ of 12.5 μmol/min/mg and *K*_m_ of 45.5 mM. *K*_m_ values using propionamide as substrate has been reported earlier from *Pseudonocardiathermophila* (7.4 mM), *Rhodococcus* sp. (1 mM), *Rhodococcuserythropolis* (1.3 mM), *Rhodococcus* sp. (2.6 mM), *Rhodococcus* sp. N-771 (34.2 mM) and *Brevibacterium* sp. (88 mM), respectively, while *V*_max_ for *Pseudomonas putida* was 0.019 (Egorova et al. 2004; Fournand et al. 1998; Park and Uhm 2008; Nawaz et al. 1994; Thiery et al. 1986; Wyndham and Slater 1986). Purified nitrile hydratase has *K*_m_ and *V*_max_ value for acrylonitrile as 40 mM and 10 μmol/min/mg, respectively. Nitrile hydratases of *Brevibacterium* sp. and *Nocardia* sp. (Kaakeh et al. 1991; Yu et al. 2006) have *K*_m_ 16.6, 16.7 and 82 mM, respectively, while *V*_max_ value for *Rhodococcus* sp. RHA1 and *Rhodococcus* sp. SHZ1 were respectively 2.8 and 12.3 μmol/min/mg (Wang et al. 2007).

### Incubation time and stability

Activities of nitrile/amide hydrolyzing enzymes were determined at different time interval (10–90 min). Nitrile hydratase and amidase gave maximum activity, respectively, 4.6 and 6.4 U/mg protein at 20 min of incubation. Amidase has half lives of 31 h, 23 h, 10 h, 4 h, 3 h and 2 h 30 min, respectively at 10, 15, 25, 35, 45 and 55 °C, while nitrile hydratase has half lives of 43, 31, 5, 3, 2.6 and 1 h, respectively at 10, 15, 25, 35, 45 and 55 °C. Both enzymes were stable at lower temperature, and increase in temperature resulted in rapid inactivation of enzyme. Amidase has half lives of 2 h 30 min at 55 °C, while amidase of *Rhodococcuserythropolis* No. 7 has a half life of 30 min at 55 °C (Park and Uhm 2008). Nitrile hydratase has half lives of 31 h at 15 °C and is more stable in comparison to nitrile hydratase of *Microbacterium imperiale* CBS 498-74 having half life of 6.5 h (Cantarella et al. 2004). Both enzymes are stable at lower temperature, increase in temperature resulted in rapid inactivation of enzyme.

### Effect of metal ions

**Fig. 6:**
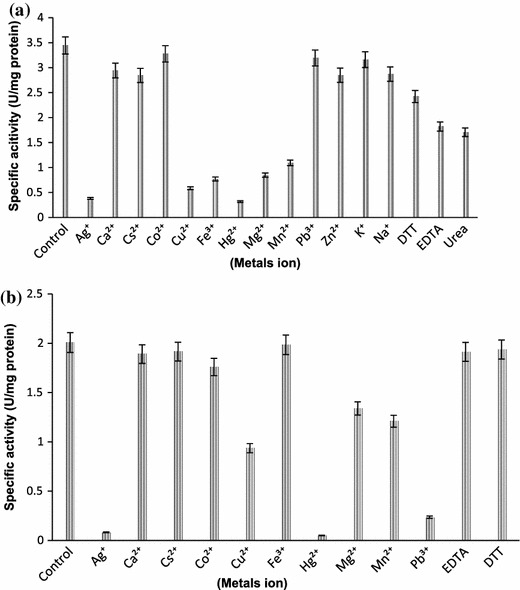
**a** Effect of metal ions on amidase activity. **b** Effect of metals ion on nitrile hydratase activity

*Alcaligenes* sp. MTCC 10674 nitrile/amide hydrolyzing enzymes were strongly inhibited by heavy metals ion. Addition of Hg^2+^, Ag^+^, Pb^2+^, Cu^2+^, Fe^2+^ and Mg^2+^ resulted into 92, 89, 88, 83, 77 and 75 % inhibition of amidase activity (Fig. 6a). In case of Nhase, addition of Hg^2+^, Ag^+^, Pb^2+^ and Cu^2+^ in reaction resulted into 97, 95, 88 and 55 % inhibition of activity, respectively (Fig. 6b). Other metal ions and chemical compounds, i.e., Ca^2+^, Cs^2+^, Co^3+^, Zn^2+^, Na^+^, K^+^, urea, EDTA and DTT showed no significant effect on nitrile hydratase and amidase activity. *Alcaligenes* sp. MTCC 10674 nitrile/amide hydrolyzing enzymes were strongly inhibited by heavy metal ions. Metal ions Hg^2+^, Ag^+^, Pb^2+^ and Cu^2+^ are strong inhibitors of nitrile hydrolyzing enzyme activity. Metal ions inhibition study revealed that enzymes contains sulfhydral (–SH), alcohol, or acid groups as part of their active sites, any reagent/metal ion which can react with these side groups of amino acid and act as an irreversible inhibitor. Heavy metals such as Ag^+^, Hg^2+^ and Pb^2+^ have strong affinities for –SH groups and inhibit activity of nitrile metabolizing enzyme *Paracoccus* sp. M-1 (Shen et al. 2012). Several amidases and nitrile hydratase enzyme have been already purified, but simultaneous purification of these two enzymes, however, appear unique which makes this purification process simple and rapid.
